# The impact of chronic low back pain on physical performance, fear avoidance beliefs, and depressive symptoms: A comparative study on Turkish elderly population

**DOI:** 10.12669/pjms.292.3196

**Published:** 2013-04

**Authors:** Emine Aslan Telci, Nesrin Yagci, Tuba CAN, Ugur Cavlak

**Affiliations:** 1 Emine Aslan Telci, PT, PhD,Asistant Professor,; 2 Nesrin Yagci, PT, PhD,Assosciate Profeessor,; 3 Tuba CAN, PT, PhD,; 4 Ugur Cavlak, PT, PhD, Professor,

**Keywords:** Low back pain, Aged, Activity limitation, Fear, Depressive symptoms

## Abstract

***Objectives:*** The purposes of this study were: (1) to show the impact of chronic low back pain (CLBP) on physical performance, fear avoidance behavior and depressive symptoms in older adults; (2) to describe the relationships between outcome measurements obtained in this study.

***Methodology: ***Ninety-one participants with or without chronic low back pain were included in this study. Only four tests in the Back Performance Scale were used to assess the physical performance of the participants. A Fear Avoidance Beliefs Questionnaire related to physical activity and the Geriatric Depression Scale were also used to examine each subject.

***Results:*** The level of performance shown by elderly adults with low back pain was worse than elders without low back pain in the sock test and the pick-up test (p < 0.05). Little correlation between the finger-to-floor test and fear avoidance behaviour related to physical activity was found (p < 0.05). There was little/poor correlation between all performance tests and depressive symptoms (p < 0.05).

***Conclusions:*** The findings indicate that CLBP decreases physical performance, but increases fear avoidance behavior and depressive symptoms in elderly adults. At the same time, it was determined that performance level of elderly adults with back pain was related especially with depressive symptoms.

## INTRODUCTION

Chronic low back pain (CLBP) is one of the most important health problems in elderly adults. The prevalence of LBP in the elderly is not accurately known but is reported to range between 13% and 49%.^1^ Patients with CLBP often report a decreased level of mobility due to pain.^2^

Mobility is a vital factor in order to be able to perform the activities of daily living. One’s state of mobility affects his/her medical condition and state of well-being.^3^ Self-report measures for related physical functions assess the perceived physical status in subjects with low back pain.^4^ Some studies show a mismatch between self-reported measurements and observed disability.^5^^,^^6^ It was stated that the tests such as muscle strength and range of motion, which are used to measure the functional level in patients with low back pain, remain incapable of determining disability in daily life as they measure determining physical impairment.^7^^,^^8^ The majority of the tests used to assess functional level have aimed to measure trunk mobility and the coordination of speed and movement, in the last few decades in particular.^8^ Some tests such as progressive isoinertial lifting test, which evaluate the functional level for patients with low back pain require suitable space, time and equipment.^9^^,^^10^ Therefore, it is important to choose the most suitable tests among various functional tests in order to determine performance level of people with CLBP.

The assessment of trunk mobility in different activities better reveals the difference between subjects with and without CLBP.^4^^,^^9^ The Back Performance Scale (BPS) is a useful instrument for determining the performance of the individuals with CLBP.^9^

The relationship between the psychological status and spinal pain has not been fully explained. One study found the incidence of depressive symptoms to be higher in community-dwelling elderly people with low back and neck pain compared to elderly people not experiencing without pain.^11^

Our main goal in this study, which was planned as a home-based study, was to determine the degree of mobility difficulty faced by older adults with CLBP during activities of daily living and to describe the impact of CLBP. We preferred to use performance-based measurements imitating the activities of daily living for this purpose.

We investigated how LBP affects the performance-based functional level, fear avoidance behavior and depressive symptoms in older adults aged 65 and over living in Denizli, Turkey. At the same time, we attempted to detect the relationship between performance-based functional level and the other outcome measurements in the participants with CLBP.

## METHODOLOGY

This is a home-based personal interview study. Participants aged 65 years or older living in their own residences were included in this study. All subjects were retired or had never worked. Inclusion criteria for the group with LBP were long-term pain (>3 months) and, for the controls (pain-free group), was having experienced no musculoskeletal pain in the last six months. All the subjects were independent with ambulation and activities of daily living. Exclusion criteria for both groups were orthopedic or neurological problems such as inflammatory rheumatic diseases, serious cardiovascular problems, visual impairment, communication problems and regular medication usage.

One hundred and twenty two patients who met the inclusion criteria were evaluated out of a total of 167 subjects aged 65 years or older. Thirty-one subjects were excluded from the study because of missing data. Finally, a total of 91 subjects (56 subjects with and 35 without CLBP) composed the population of this study.

This was a single-blind study. An examiner noted the demographics and completed the questionnaires. Demographic data were recorded with a prepared questionnaire (Table-I). All performance tests were supervised by another examiner. The second examiner did not know to which group the subject’s belonged. Ethics committee approval for the study was obtained from Ethics Committee of Clinical Researches, Pamukkale University (B.30.2.PAU.0.20.05.09/84).

The five BPS physical performance tests which involve trunk mobility and combined movement are useful to assess activity limitations in patients with back pain. These tests, which entail harmonious movement of body and lower limbs, begin from different starting positions such as standing and require different angular values of knees.^4^^,^^9^ We used only the following four tests (the sock test, the pick-up test, the roll-up test and the finger-to-floor test) which could be easily performed. The lifting test entails lifting a box (1.35 kg; 0.36× 0.36× 0.25 cm), in which a sandbag with a weight of 5 kg was placed, from floor to the table (at a height of 76 cm.) repetitively for one minute.^9^ This test was not included due to the difficulty of carrying materials required for the test.

Each BPS test was scored from 0 to 3 over the four-point ordinal scales according to the observed physical performance level.^9^ Pain intensity was evaluated with the 10 cm Visual Analog Scale (VAS) (0= no pain; 10= unbearable pain). Subjects were asked to indicate their general pain intensity on the scale.

The fear avoidance beliefs were evaluated with The Turkish version The Fear Avoidance Beliefs Questionnaire (FABQ) was developed by Waddel et al.^12^^,^^13^ We used only the physical activity section from the FABQ since none of the subjects in our study were employed. The physical activities score was classified as low fear (0-14 points) or high fear (15 points or more) according to the median split value defined by Burton et al.^14^

The Turkish version of the Geriatric Depression Scale (GDS) was used to determine depression in older adults.^15^^,^^16^ When the cut-off point was accepted as 14 (0-13 depressive signs absent; 14 and over depressive signs present) for this scale, sensitivity and specificity were found as 0.90, 0.94, respectively.^17^ Therefore, the cut-off point was also accepted as 14 for this study.

***Statistical Analysis: ***Descriptive data were presented as mean ± standard deviation (SD) or as a percentage (%). The Kolmogorov-Smirnov test was used to examine the normality of the data. Student’s t test was used to compare demographic variables and FABQ-PA and GDS scores between older adults with/without low back pain. The chi-square test was used to compare 4 tests of the BPS between the two groups. The correlations between the scores of performance tests, self-report questionnaires, pain intensity and pain duration were determined using by Spearman correlation test. The correlations were interpreted as follows: 0.25 or less little if any relationship, 0.26–0.49 poor relationship, 0.50–0.69 moderate relationship, 0.70–0.89 strong relationship, 0.90–1.00 very strong relationship.^18^ P values less than 0.05 were considered statistically significant.

## RESULTS

There were 37 females (66.1%) and 19 males (33.9%) with CLBP. There were 14 females (40%) and 21 males (60%) in the controls without LBP. There were no differences between the groups in terms of age, weight, height, BMI and years in education (Table-I) (p>0.05). The mean pain intensity was 5.40 ± 1.93 cm (range; 2-10) for older adults with CLBP. The other pain-related information for the CLBP group can be seen in Table-I.

The level of performance shown by older adults with lower back pain was worse than elders without lower back pain in the sock test and the pick-up test (p<0.05). There were no significant differences in terms of finger-to- floor test and the roll- up test between two groups (p> 0.05). The scores of fear avoidance beliefs related to physical activity and depression were also found to be higher in older adults with CLBP (p<0.05) compared free-pain older adults (Table-II).

No correlation was found between the individual test scores and pain severity, and pain duration in the group with CLBP. Little correlation was found between the finger- to- floor test and fear avoidance behavior associated with physical activity (p<0.05). Little/ poor correlation was found between the individual test scores and depressive symptoms (p<0.05) (Table-III).

**Table-I:** Demographics of the participants by groups.

*Variables*	*Group I* *With low back pain (n=56)* *Mean ± SD*	*Group II* *Without low back pain (n=35)* *Mean ± SD*	p*
Age (year)	71.21 ± 6.40	72.51 ± 7.34	0.37
Weight (kg)	73. 20 ± 12.8	75.23 15.87	0.51
Height (cm)	1.65 ±0.90	1.67 ±0.8	0.17
Body Mass Index (kg/m²)	26. 86 ± 3.87	26.89 ± 4.08	0.98
Education in year	3.23 ± 3.88	4.14 ± 11.82	0.59
Pain intensity (VAS, cm)	5.40 ± 1.93	-	-
Pain duration (months)	82.29 ±92.94	-	-

*Student’s t test*

**Table-II:** Comparison of the Groups in terms of Physical Performance Tests, Fear Avoidance Behavior Related Physical Activity and Depressive Symptoms.

	*Group I*	*Group II*	
	*With LBP Group (n=56)*	*Without LBP Group (n=35)*	
	*N(%)*	*N(%)*	*P*
Tests			
Sock			
0	10 (17.9)	19 (54.3)	
1	22 (39.3)	8 (22.9)	0.001
2	17 (30.4)	3 (8.6)	
3	7 (12.5)	5 (14.3)	
Pick-up			
0	10 (17.9)	16 (45.7)	
1	19 (33.9)	10 ( 28.6)	0.031
2	22 (39.3)	8 (22.9)	
3	5 (8.9)	1 (2.9)	
Finger-to- floor			
0	8 (14.3)	5 (14.3)	
1	23 (41.1)	21 (60.0)	0.300
2	18 (32.1)	6 (17.1)	
3	7 (12.5)	3 (8.6)	
Roll-up			
0	4 (7.1)	5 (14.3)	
1	19 (33.9)	19 (54.3)	
2	20 (35.7)	7 (20.0)	0.089
3	13 (23.2)	4 (11.4)	
	Mean ± SD	Mean ± SD	P
FABQ-PA	16.82 ± 4.38	13.51 ± 6.48	0.005
GDS	14.12 ± 6.01 / 14*	10.51 ± 5.80 / 14*	0.006

*Cut-off point; FABQ-PA Fear Avoidance Beliefs Questionnaire- Physical Activity;

*GDS Geriatric Depression Scale*

**Table-III:** Correlation among performance tests, pain intensity, pain duration, fear avoidance beliefs related physical activity, and depressive symptoms.

	*Pain intensity* *(VAS, cm)*	*Pain duration (month)*	*FABQ-PA*	*GDS*
Sock test	0.249	-0.153	-0.103	0.240*
Pick-up test	0.198	-0.070	0.168	0.285*
Roll-up test	0.181	-0.206	-0.094	0.247*
Fingertip-to-floor test	0.058	-0.031	-0.242*	0.299*

*p<0.05; FABQ-PA Fear Avoidance Beliefs Questionnaire- Physical Activity;*

*GDS Geriatric Depression Scale.*

## DISCUSSION

Our results point out that back pain among older adults influence specific functional activities about activities of daily living. Moreover it was determined that performance level among older adults with back pain is not related with pain and fear avoidance behavior (except to finger-to-floor) but related with depressive symptoms at low level. 

 Our results obtained from the sock test and the pick- up performance tests are in conformity with literature.^19^^,^^20^ On the other hand, we concluded that there is no significant difference between older adults with and without pain in the finger-to-floor and the roll-up tests. The finger-to-floor test is a test that entails shoulder and hip movement in addition to body mobility.^21^ At the same time, the roll-up test, which requires adopting a sitting position, is related to abdominal muscle strength.^22^ Flexibility and strength evaluation was not carried out as part of our study. There is a possibility that similar muscle strength and flexibility values may have affected outcomes in both groups.

There are a few studies which analyse the relation between performance level, fear avoidance behaviour and depressive symptoms in patients with low back pain.^6^^,^^23^^,^^24^ Reneman et al. found that the relationship between pain, pain-related fear and functional performance level is generally poor or not significant. Investigators reported that patients performed the functional tests despite their pain. ^6^ Geisser et al. determined that there is a meaningful relation between floor to waist lifting section and fear of Progressive Isoinertial Lifting Evaluation which is used in order to evaluate performance but there is not a meaningful relation between waist to shoulder section and fear.^10^ This result demonstrates that the applied test battery affects pain-related fear behavior. Magnussen et al, determined that there is no correlation between fear in low back pain and the total score of BPS. Authors explained this situation in the way that fear associated with low back pain is associated with the future rather than the current situation but BPS is not predictive.^4^ Our results show that despite low correlation, fear behaviour may occur due to physical activity and mental state influences performance level. Previous literature and our study show that many factors such as low educational level of the study population, brevity of the implemented tests, high motivation and chosen tests influence results.^2^^,^^6^^,^^11^^,^^25^

As far as we know, this is the first home-based study which was conducted in Turkey in this field which is a strength of our study. Moreover in this study we have analyzed the relation between performance based functional tests, fear avoidance behaviour and depressive symptoms in older adults with low back pain. This makes our study quite important.

***Limitation of the study: ***There are some limitations of this study. The most important limitation is excluding lifting test which is the most challenging item of The Back Performance Scale. On the other hand, that we obtained meaningful results in only two tests among four tests we have used which made it difficult for us to make comment about the effectiveness of BPS in determining activities of daily living and relative performance level. The fact that the test battery which we used only evaluates movements in the sagittal plane is regarded as the limitation of our study. On the other hand, Magnussen et al. found a high correlation between BPS and the Der Funktionsfragenbogen Hannover Questionnaire, which contains twisting and side bending works in addition to items similar to four items of BPS. Therefore, they surmmarized that evaluating movements in different planes in addition to BPS is not necessary in low back pain.^4^

## CONCLUSIONS

It is quite important to determine the level of inability in performing functional activities of older adults with back pain in their own home environment where they spend most of the time. For this aim, it would bring more accurate results to evaluate people in their own living environment based on observation. However, since only two out of four tests we have used in measurement have significant results show that more comprehensive studies which are suitable for the daily activities of older adults with back pain in home environment should be carried out. This helps us to understand how the CLBP interfers with daily living activities. Further studies are needed to confirm our finding.

## References

[1] Bressler HB, Keyes WJ, Rochon PA, Badley E (1999). The prevalence of low back pain in the elderly A systematic review of the literature. Spine.

[2] Weiner DK, Haggerty CL, Kritchevsky SB, Harris T, Simonssick EM, Nevitt M (2003). How does low back pain impact physical function in independent, well-functioning older adults? Evidence from the Health ABC Cohort and implications for the future. Pain Med.

[3] Frank JS, Patla AE (2003). Balance and mobility challanges in older adults: implications for preserving community mobility. Am J Prev Med.

[4] Magnussen L, Strand LI, Lygren H (2004). Reliability and validity of the Back Performance Scale: Observing activity limitation in patients with back pain. Spine.

[5] Wand BM, Chiffelle LA, O'Connell NE, McAuley JH, Desouza LH (2010). Self-reported assessment of disability and performance-based assessment of disability are influenced by different patient characteristics in acute low back pain. Eur Spine J.

[6] Reneman MF, Schiphorts Preuper HR, Kleen M, Geertzen JH, Dijkstra PU (2007). Are pain intensity and pain related fear related to functional capacity evaluation performances of patients with chronic low back pain. J Occup Rehabil.

[7] Deyo RA (1988). Measuring the functional status of patients with low back pain. Arch Phys Med Rehabil.

[8] Caporaso F, Pulkovski N, Sprott H, Mannion AF (2012). How well do observed functional limitations explain the variance in Roland Morris scores in patients with chronic non-specific low back pain undergoing physiotherapy. Eur Spine J.

[9] Strand LI, Moe-Nilssen R, Ljunggren AE (2002). Back Performance Scale for the assessment of mobility-related activities in people with back pain. Phys Ther.

[10] Geisser ME, Haig AJ, Theisen ME (2000). Activity Avoidance and Function in Persons with Chronic Back Pain. J Occup Rehabil.

[11] Fernández-de-las-Penãs C, Hernández-Barrera V, Alonso-Blanco C, Palacios-Ceña D, Carrasco-Garrido P, Jiménez-Sánchez S (2011). Prevalence of neck and low back pain in community-dwelling adults in Spain. A population-based national study. Spine.

[12] Waddell G, Newton M, Henderson I, Somerville D, Main CJ (1993). A Fear-Avoidance Beliefs Questionnaire (FABQ) and the role of fear-avoidance beliefs in chronic low back pain and disability. Pain.

[13] Korkmaz N, Akinci A, Yorukan S, Surucu HS, Saracbasi O, Ozcakar L (2009). Validation and reliability of the Turkish version of the fear avoidance beliefs questionnaire in patients with low back pain. Eur J Phys Rehabil Med.

[14] Burton AK, Waddell G, Tillotson KM, Summerton N (1999). Information and advice to patients with back pain can have a positive effect. A randomized controlled trial of a novel educational booklet in primary care. Spine.

[15] Yesavage JA, Brink TL, Rose TL, Lum O, Huang V, Adey M (1982-1983). Development and validation of a geriatric depression screening scale: A preliminary report. J Psychiatr Res.

[16] Ertan T, Eker E (2000). Reliability, validity, and factor structure of the geriatric depression scale in Turkish elderly: Are there different factor structures for different cultures. Int Psychogeriatr.

[17] Sagduyu A (1997). The Geriatric Depression Scale A Reliability and Validity Study in Comparison with Hamilton Rating Scale for Depression. Turk Psikiyatri Dergisi.

[18] Munro BH, Visintainer MA, Batten Page E (1986). Statistical methods for health care research.

[19] Simmonds MJ, Olsın SL, Hussein T, Lee CE, Novy D, Radwan H (1998). Psychometric characteristics and clinical usefulness of physical performance tests in patients with low back pain. Spine.

[20] Teixeira da Cunha-Filho, Lima FC, Guimarães FR, Leite HR (2010). Use of physical performance tests in a group of Brazilian Portuguse-speaking individuals with low back pain. Physiother Theory Pract.

[21] Michel A, Kohlmann T, Raspe H (1997). The association between clinical findings on physical examination and self-reported severity in back pain. Results of a population- based study. Spine.

[22] Childs JD, Teyhen DS, Benedict TM, Morris JB, Fortenberry AD, McQuenn RM (2009). Effects of sit-up trainning versus core stabilization exercises on sit-up performance. Med Sci Sports Exerc.

[23] Al- Obaidi SM, Al-Zoabi B, Al-Shuwaie N, Al- Zaabie N, Nelson RM (2003). The influence of pain- related fear and disability beliefs on walking velocity in chronic low back pain. Int J Rehabil Res.

[24] Crombez G, Vlaeyen JW, Heuts PH, Lysens R (1999). Pain-relatedfear is moredisabling than pain itself: Evidence on the role of pain-relatedfear in chronic back pain disability. Pain.

[25] Swinkels-Meewisse IE, Roelofs J, Verbeek AL, Oosterndorp RA, Vlayen JW (2003). Fear of movement/(re)injury, disability and participation in acute low back pain. Pain.

